# ATACAmp: a tool for detecting ecDNA/HSRs from bulk and single-cell ATAC-seq data

**DOI:** 10.1186/s12864-023-09792-6

**Published:** 2023-11-10

**Authors:** Hansen Cheng, Wenhao Ma, Kun Wang, Han Chu, Guangchao Bao, Yu Liao, Yawen Yuan, Yixiong Gou, Liting Dong, Jian Yang, Haoyang Cai

**Affiliations:** Center of Growth, Metabolism, and Aging, Key Laboratory of Bio-Resources and Eco-Environment, College of Life Sciences, Sichuan University, No.29 Wangjiang Road, Chengdu, Sichuan 610064 China

**Keywords:** ecDNA, ATAC-seq, Cancer genome, Intratumor heterogeneity

## Abstract

**Background:**

High oncogene expression in cancer cells is a major cause of rapid tumor progression and drug resistance. Recent cancer genome research has shown that oncogenes as well as regulatory elements can be amplified in the form of extrachromosomal DNA (ecDNA) or subsequently integrated into chromosomes as homogeneously staining regions (HSRs). These genome-level variants lead to the overexpression of the corresponding oncogenes, resulting in poor prognosis. Most existing detection methods identify ecDNA using whole genome sequencing (WGS) data. However, these techniques usually detect many false positive regions owing to chromosomal DNA interference.

**Results:**

In the present study, an algorithm called “ATACAmp” that can identify ecDNA/HSRs in tumor genomes using ATAC-seq data has been described. High chromatin accessibility, one of the characteristics of ecDNA, makes ATAC-seq naturally enriched in ecDNA and reduces chromosomal DNA interference. The algorithm was validated using ATAC-seq data from cell lines that have been experimentally determined to contain ecDNA regions. ATACAmp accurately identified the majority of validated ecDNA regions. AmpliconArchitect, the widely used ecDNA detecting tool, was used to detect ecDNA regions based on the WGS data of the same cell lines. Additionally, the Circle-finder software, another tool that utilizes ATAC-seq data, was assessed. The results showed that ATACAmp exhibited higher accuracy than AmpliconArchitect and Circle-finder. Moreover, ATACAmp supported the analysis of single-cell ATAC-seq data, which linked ecDNA to specific cells.

**Conclusions:**

ATACAmp, written in Python, is freely available on GitHub under the MIT license: https://github.com/chsmiss/ATAC-amp. Using ATAC-seq data, ATACAmp offers a novel analytical approach that is distinct from the conventional use of WGS data. Thus, this method has the potential to reduce the cost and technical complexity associated ecDNA analysis.

**Supplementary Information:**

The online version contains supplementary material available at 10.1186/s12864-023-09792-6.

## Background

**Fig. 1:**
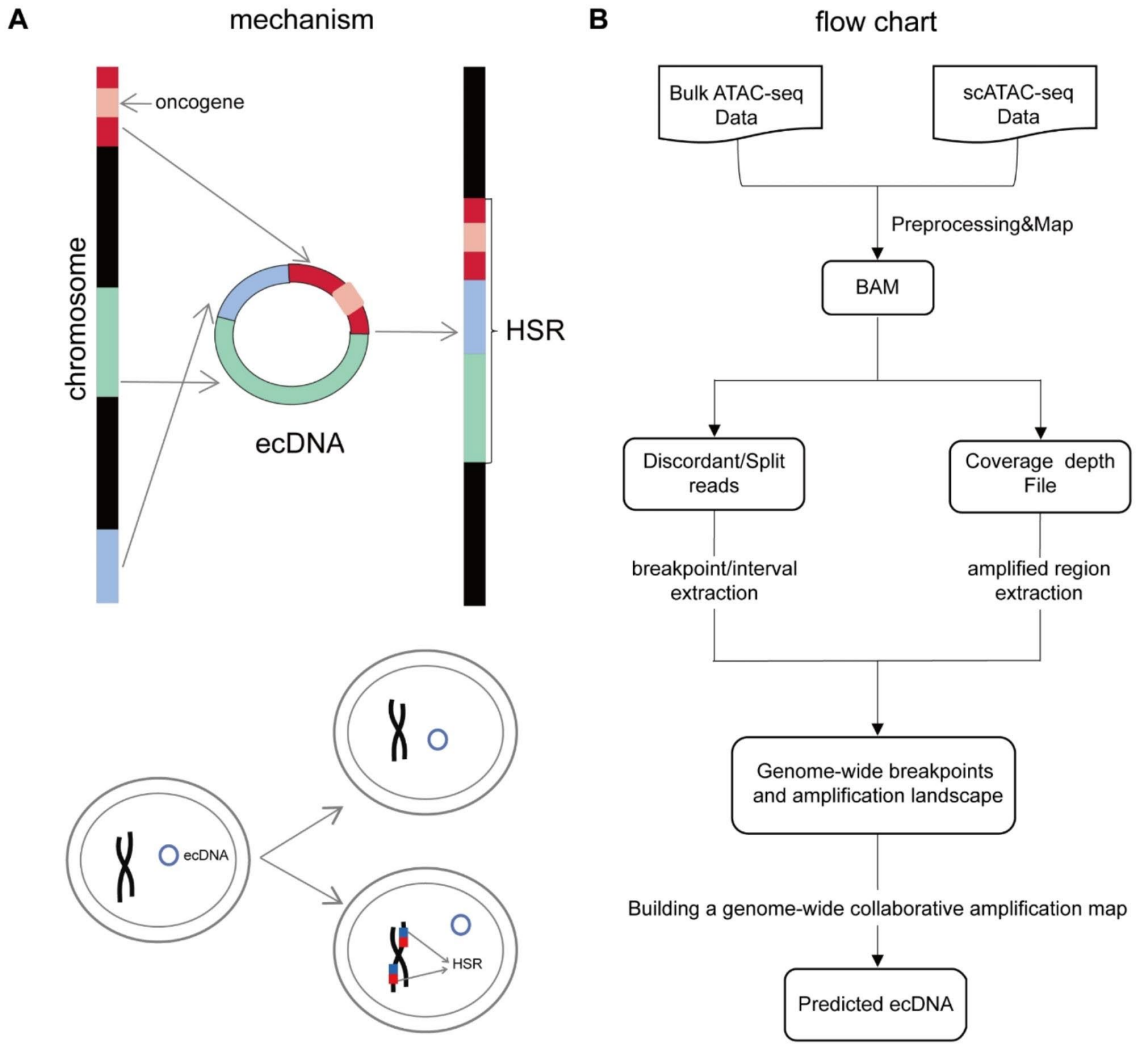
Schema of ecDNA/HSR formation and the pipeline of ATACAmp. (**A**) Fragments on the chromosome form ecDNA, which is then reintegrated into the chromosome to form HSR. (**B**) The ATACAmp analysis starts from the BAM file, extracts and processes abnormal reads to determine the breakpoint position, and then considers the sequencing depth near the breakpoint to determine the amplification region

Extrachromosomal DNA (ecDNA), a specific type of circular DNA, is found in tumor cells [1–3]. EcDNAs comprise DNA fragments ranging from hundreds of kilobases to several megabases that originate from one or multiple chromosomal regions [4]. Of note, ecDNAs lack a centromere and often harbor sequences that confer cell survival advantages [5]. Through unequal division, ecDNAs can rapidly accumulate in daughter cells (Fig. 1A), and under unfavorable conditions, they can integrate into chromosomes and form homogeneously staining regions (HSRs) [6]. An HSR is a tumor-specific structure that contains areas that display uniform brightness after Giemsa staining, rather than the bright and dark interlacing regions of normal chromosomal regions. These regions have been shown to amplify oncogenes [7].

EcDNA/HSR detection is challenging owing to its large size and complex composition. The technical limitations of DNA extraction make it difficult to directly extract complete DNA fragments sized > 100 Kb. Moreover, unlike gene copy number detection, ecDNA/HSR detection algorithms must consider the synergistic amplification of different fragments rather than a focal amplicon. Some algorithms detect ecDNA/HSR using whole genome sequencing (WGS) data. For example, AmpliconArchitect (AA) has been specifically designed to detect large extrachromosomal circular DNA in tumor cells using short-read WGS data [8]. However, AA prediction results often include false-positive regions, necessitating manual annotation and selection to obtain a more accurate ecDNA sequence. Experimental methods are now available to obtain information about ecDNA. CRISPR-Cas can accurately determine the sequence and epigenetic modifications of ecDNA via the following steps: ecDNA cutting using the CRISPR-cas9 method, fragment separation via pulsed-field electrophoresis, and sequencing with second- or third-generation sequencing methods [9]. However, CRISPR-Cas requires prior knowledge of the ecDNA sequence and a complex experimental protocol. Therefore, more effective methods are needed to study ecDNA/HSRs.

Although ATAC-seq was designed to detect open chromatin regions, it is suitable for identifying ecDNA/HSRs [10]. Many studies have found that chromatin accessibility in ecDNA/HSRs is higher than that in normal chromosomal regions [11, 12]. This may be attributed to lower compression in ecDNA/HSRs as they are formed outside of chromosomes. The accumulation of intracellular ecDNA is detected as an increased copy number of the corresponding genomic segments in WGS data and as a contiguous high signal in ATAC-seq data. In recent years, the scATAC-seq technology has emerged, facilitating chromatin accessibility detection at the single-cell scale and expanding the existing knowledge on the heterogeneity of chromatin accessibility between cells [13]. ATAC-seq data is primarily used to study the accessibility of genomic regions, despite ATAC-seq also being genome-wide DNA-seq data [14]. The ATAC-seq of tissues or single cells can provide information about some types of genomic variation in the sample.

Although circle-hunter, a pipeline that helps predict ecDNA using ATAC-seq data, has appeared in the recent past, but it cannot be used for single-cell ATAC-seq data [15]. Therefore, a new tool, called ATACAmp, was developed herein for detecting ecDNA/HSRs. ATACAmp exploits the unique characteristics of ecDNA/HSRs in ATAC-seq data and enables the detection of these genomic variations in a more streamlined and efficient manner (Fig. 1B). By taking the advantage of the potential of ATAC-seq data in genomic variant detection, researchers can gain a deeper understanding of the complex interplay between chromatin accessibility and genomic structure in the context of certain diseases, such as cancer.

### Implementation

ATACAmp is currently supported on the Linux system only, as it relies on the availability of the Pysam package. Before using ATACAmp, users need to install the Python modules of Pysam, Multiprocessing, Argparse, Subprocess, and Interval. Users can obtain information about the relevant parameter settings using the “-h” parameter in the ATACAmp program.

To use ATACAmp, users should first pre-process their sequencing data, which involves quality control, alignment, sorting, and filtering. High-quality reads are critical for obtaining reliable results. Once the sorted BAM file and the corresponding BAI index are obtained, users can operate ATACAmp. Users can optionally utilize software such as Samblaster to identify abnormal reads, and ATACAmp permits users to initiate analysis from different stages. This can be specified using the “mode” parameter, which indicates whether to start from the BAM file or from abnormal reads.

Users have the option to choose between single-cell mode and bulk mode. The single-cell mode provides the barcodes of supported cells for each ecDNA/HSR region. ATACAmp employs multiprocessing to accelerate calculations, and users can specify the number of cores to be used via the “threads” parameter. It is recommended to set the thread parameter to 24 to attain the maximum processing speed.

Users can upload a corresponding GTF file for annotating the amplification region. The threshold value for detecting abnormal read segments is user-defined and recommended to be set at 1000. The threshold value can be adjusted as per the specific library preparation methods. While a lower threshold value will generally yield more abnormal read segments, necessitating additional subsequent calculations, an excessively high threshold value may lead to the loss of some abnormal read segment information. A minor adjustment to the threshold value, however, will not significantly affect the results.

Users have the flexibility to set the interval size for the extended amplification region introduced in the Methods section; the default interval size is set to 1000. A smaller interval can detect finer amplification regions but will take longer to analyze. After testing, it was determined that an interval size of 1000 bp is optimal.

During the run, ATACAmp provides updates on the progress and time taken for the analysis. For bulk data, executing a BAM file with 100 million lines(about 2.5Gb) typically takes approximately 30 min. For single-cell data, the processing time is approximately 1.5-fold longer than that for bulk data with the same amount of data.

The results of the run comprise multiple files, including a BAM file that contains abnormal read segments and a file that shows the breakpoint locations, with final information on the amplification regions. These files are provided to enable users to customize their analysis.

## Results

### ATACAmp results for bulk-cell ATAC-seq dataset

**Fig. 2:**
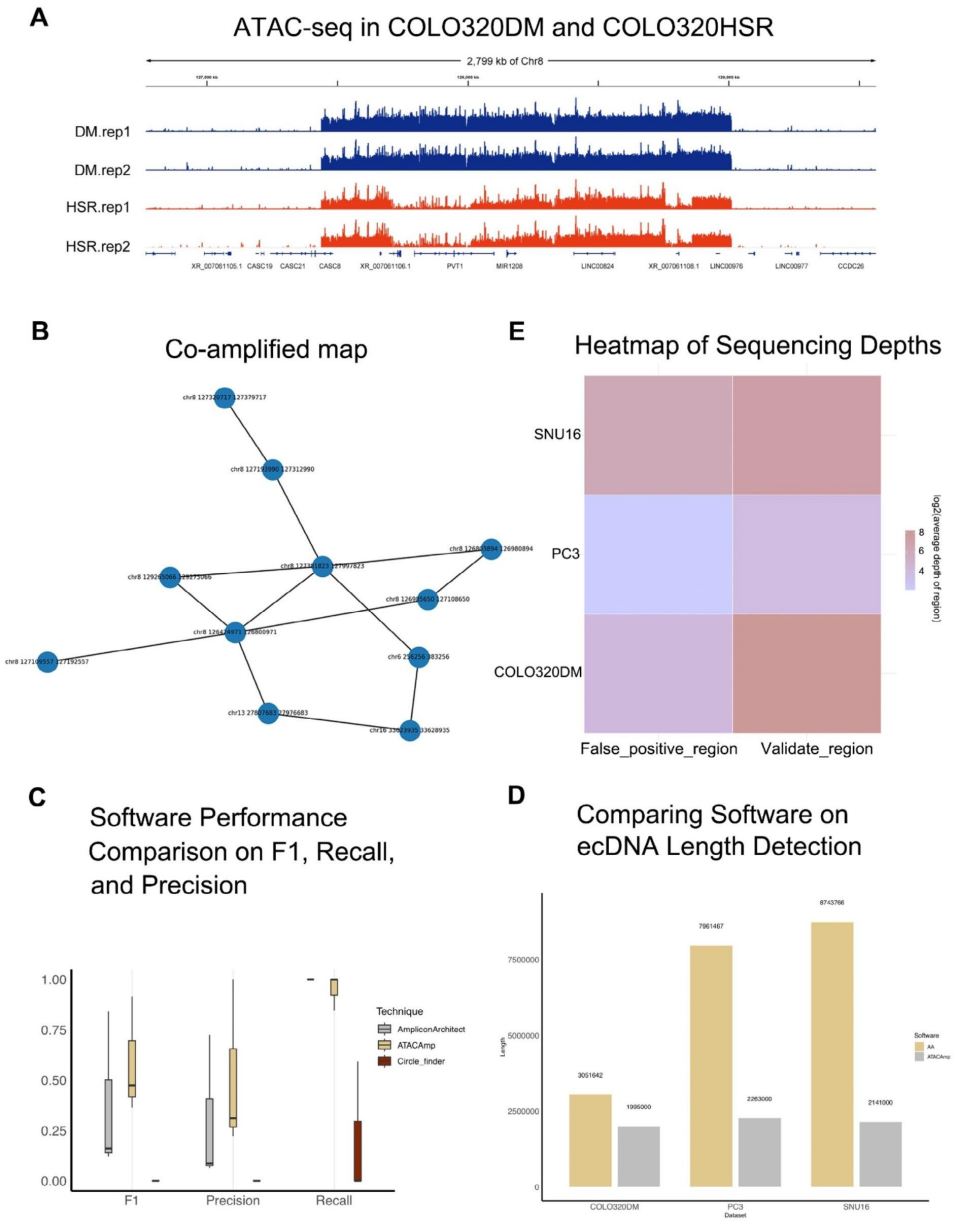
ATAC-seq features of ecDNA/HSR and performance of ATACAmp. (**A**) ATAC-seq profile of ecDNA regions in the COLO320DM and COLO320HSR cell line. (The upper two tracks are ecDNA-forming regions in the COLO320DM cell line and the lower two tracks are HSR-forming regions in the COLO320HSR cell line) (**B**) The links of the highest scoring co-amplified regions in the COLO320DM cell line. (**C**) The performances of different ecDNA calling softwares. (**D**) Comparison of total length of ecDNA detected by different software. (**E**) Heatmap of Sequencing Depths for False-Positive and Validated AA Regions

In samples with ecDNA or HSRs, variant structures will be expressed as continuous high signal areas in ATAC-seq analysis (Fig. 2A). The ATAC-seq MACS results of HSRs in the COLO320HSR cell line and ecDNA regions in the COLO320DM cell line indicated that these regions had high chromatin accessibility. Of note, such contiguous open regions that span millions of base pairs are not observed in normal cells [4]. Bulk ATAC-seq data for COLO320DM, SNU16, and PC3 cells were then examined (Table S1). The fragment length threshold used to identify discordant reads was set to 1000 bp, and the range of merging neighboring breakpoints was set to 1000 bp on both sides. All other parameters were set to default. The detected amplified regions and affected genes are shown in supplementary Table S2.

Figure 2B shows that ATACAmp detected the highest scoring co-amplified regions in COLO320DM. “Co-amplified” refers to a region of the genome that undergoes simultaneous amplification because of some cellular event or mechanism, which in this paper is a general term for ecDNA and HSRs. In the current study, co-amplified regions were genomic regions that amplified together with other regions, suggesting a common mechanism or cause for their amplification. The score is determined by abnormal reads in these regions. In general, the higher is the abnormal mapping of reads, the higher is the score. Each node in the graph is an independent amplified region, and the connection between regions is determined via breakpoint pairs. All the interconnected regions have high sequencing depth and are therefore considered as co-amplified regions.

In the PC3 cell line, ATACAmp accurately identified all experimentally validated ecDNA/HSRs. In the COLO320DM and SNU16 cell lines, however, ATACAmp detected 84.34% and 99.92% of the experimentally validated ecDNAs, respectively. These results indicate that ATACAmp has a high sensitivity (Table S3).

AA is currently the most accurate software for predicting ecDNA and HSRs in tumor cells. WGS data have the advantage of containing the entire genome information, enabling AA to detect break-fusion-bridge and complex rearrangements as well as predict ecDNA/HSRs. In ATAC-seq, the Tn5 transposase has difficulty inserting itself into the condensed heterochromatin region, thereby reducing the interference of a large section of the regional sequence on the linear chromosome.

The accuracy and recall of three methods, i.e., AA, ATACAmp, and Circle-finder, were assessed using experimentally validated ecDNA regions from three cell lines as reference standards [16]. The results, as depicted in Fig. 2C, indicated that AA, with WGS data, achieved a predicted recall of 1 for ecDNA regions. By contrast, ATACAmp exhibited a slightly lower recall of 0.948 using ATAC-seq data, indicating a potentially small number of missed amplified regions. Nevertheless, this still represents a high score. Of note, ATACAmp demonstrated significantly higher precision than AA. As a result, the final combined F1 score of ATACAmp surpassed that of AA. As mentioned above, the intrinsic characteristics of ecDNA because of its open nature render ATAC-seq data more suitable for the precise identification of ecDNA regions. Meanwhile, the results also showed that Circle-finder did not detect experimentally validated ecDNA regions in both PC3 and COLO320DM cells and only partially predicted them in SNU16 cells. However, there were significant false positive regions, which almost masked the real ecDNA region information, which can be attributed to the Circle-finder algorithm being based on split reads without considering the copy number. We believe that Circle-finder is more suitable for eccDNA detection of a few hundred bp to a few Kb.

These results support the rationale that compared with experimental data, AA identifies a larger region of amplification in the same cell line, including false positives not present on the ecDNA (Fig. 2D and E). By comparison, the ecDNA regions from ATACAmp prediction overlap more with experimental validation data and annotate all oncogenes.

### ATACAmp results for single-cell ATAC-seq dataset

The ATAC-seq technology has been widely used for single cells, but few tools are available to analyze their data. ATACAmp supports the analysis of single-cell ATAC-seq data, thereby offering researchers with more options for subsequent analysis, including identifying and annotating ecDNAs to cell populations. Of note, ATACAmp can extract the barcode of the supporting cells with the amplified region.

Single-cell ATAC-seq data for COLO320DM cells were examined (Table 1). The fragment length threshold used to identify discordant reads was set to 1000 bp, and the range of merging neighboring breakpoints was set to 1000 bp on both sides.

**Table 1:** Composition of ecDNA detected in single cell ATAC data from COLO320DM cell line

Chr_name	Start_site	End_site	Length	Gene
16	33,344,230	33,431,230	87,000	TP53TG3,TP53TG3C
				LOC105369266
				TP53TG3F,TP53TG3E
				TP53TG3B,LOC102723655
8	130,277,390	130,287,390	10,000	ASAP1
8	128,206,232	128,325,232	119,000	
8	127,997,937	128,120,937	123,000	MIR1207,PVT1
				MIR1206
16	33,293,089	33,296,089	3000	
6	256,190	383,190	127,000	DUSP22
8	128,121,842	128,204,842	83,000	MIR1208
16	32,296,519	32,301,519	5000	
8	127,816,213	127,993,213	177,000	PVT1,TMEM75,MIR1205
16	32,349,447	32,371,447	22,000	
8	135,017,057	135,020,057	3000	
16	33,239,097	33,242,097	3000	
8	127,437,402	127,813,402	376,000	PVT1,MYC,CASC11,CASC8
				MIR1204
22	24,665,296	24,668,296	3000	
8	128,332,468	128,392,468	60,000	
15	20,459,784	20,462,784	3000	HERC2P3
8	128,393,562	129,010,562	617,000	LINC00976,LINC00824
13	28,381,083	28,555,083	174,000	FLT1

**Fig. 3:**
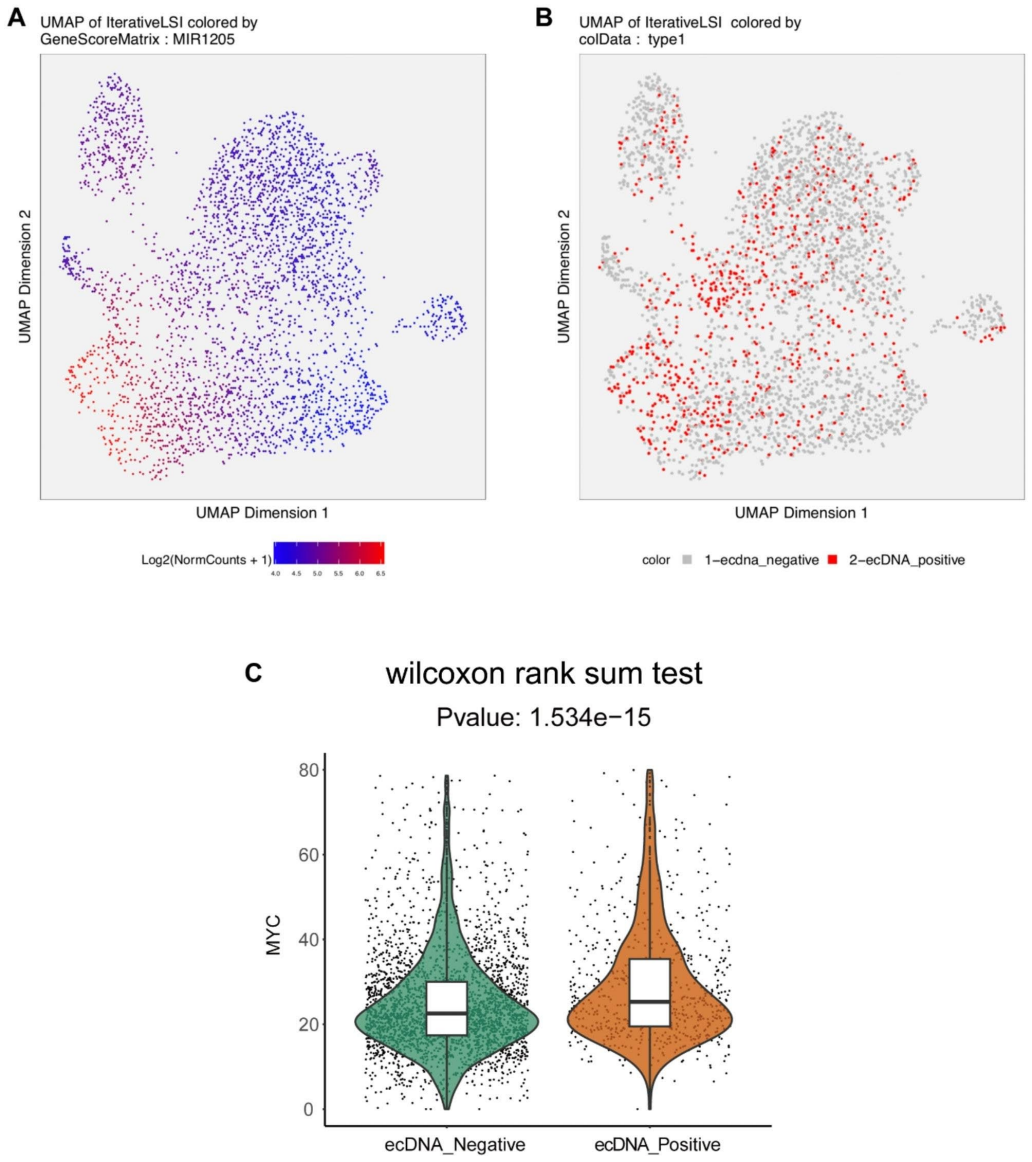
MYC expression heterogeneity in the colo320dm cell line. (**A**)The distributions of ecDNA positive cells in two-dimensional UMAP space. (**B**) MYC accessibility scores were visualized on the ATAC–seq UMAP, showing cell-level heterogeneity in MYC ATAC-seq signals in COLO320DM. (**C**) Differences in accessibility scores of MYC gene between ecDNA positive and negative cells

ATACAmp extracted the barcode of supporting cells containing the amplified regions obtained from the ATACAmp assay of COLO320DM cells. The corresponding cells in the scATAC data of COLO320DM with MYC gene expression were labeled, and the data are displayed in Fig. 3A. The distribution of the MYC gene scores is displayed in different colors, with cells carrying ecDNA containing MYC having higher MYC scores (Fig. 3B C). This suggests that cells in the COLO320DM cell line harbor different ecDNAs and that ATACAmp can identify cells that carry a certain co-amplified region.

## Discussion

Current research suggests that ecDNA is derived from chromosomal DNA, although the exact mechanism of its formation remains unclear [4]. It may either result from a continuous segment of chromosome breaking and cyclizing to form circular DNA or from chromothripsis or other events that produce various DNA fragments that are subsequently joined and cyclized to form circular DNA [17, 18]. The latter is more common in tumor cells and likely contains a higher proportion of ecDNA, as it is too large to be formed from a single region and because ≥ 100 kb linear DNA is prone to breakage.

In accordance with this property, structural variations are frequently identified by comparing WGS data to the reference genome data. ecDNA, formed by a specific region, manifests in the comparison results as a read segment that spans two regions. This suggests that the read segment contains breakpoints, whereas the other read segment of the pair is located near the junction of the circular DNA. Owing to the substantial physical distance between these regions on the reference genome, the two read segments of such read segment pairs have abnormal distances. AA can detect ecDNA from WGS data, as it also uses the whole genome copy number variation information to determine amplification regions based on considering abnormal read segments described above and includes structural variation integration to determine amplification types. However, because WGS data are subject to several interfering factors, the false-positive regions obtained are large and need to be judged by a researcher with relevant experience. In the present study, ATACAmp was developed to predict ecDNA/HSRs in ATAC-seq data by taking advantage of the open DNA feature of the ecDNA/HSR itself and demonstrated its similar sensitivity and lower false-positive rate than AA with regard to experimental validation data. Users can adjust the parameters as needed to get the desired results, such as modifying the length threshold for determining discordant reads, initial interval size for detecting the sequencing depth, and step size of each moving interval.

ATACAmp has good analysis capabilities for single-cell data, and cell populations harboring different ecDNA can be obtained for subsequent analysis. The MYC gene was selected for analysis owing to its well-established role as an oncogene and its presence on the ecDNA of COLO320DM cells. Furthermore, other genes on ecDNA were randomly selected, and it was observed that all these genes exhibited increased expression in ecDNA-positive cells (Figure S1, S2). These results are consistent with previous studies that showed gene amplification and high expression on ecDNA. Moreover, these findings suggest the presence of heterogeneity in the ecDNA content among different cell lines. The use of ATACAmp can help researchers consolidate the scope of ecDNA investigations.

## Conclusion

The current lack of research methods remains a significant concern for researchers studying ecDNA. ATACAmp, using ATAC-seq data, offers a novel analytical approach that is distinct from the conventional use of WGS data. This approach has the potential to reduce the cost and technical complexity associated with studying ecDNA. ATACAmp was validated in PC3, COLO320DM, and SNU16 cell lines where ecDNA sequences have been experimentally determined, and it showed a high recall similar to traditional predictions using WGS data, with a higher precision. The widespread adoption of single-cell ATAC-seq, compared with single-cell genomes, holds promise for investigating ecDNA within smaller cell populations.

Despite its advantages, ATACAmp has several limitations. These include the lack of validation with simulated data and the need for further clinical sample testing. In addition, certain blacklisted regions of the genome are temporarily unfiltered, possibly resulting in the identification of false-positive regions. In the future, blacklisted regions and conserved chromatin open regions will be integrated into ATACAmp to improve its accuracy. Furthermore, more data will be gathered and analyzed to optimize this tool for improved performance.

## Methods

### Data collection

The bulk ATAC-seq and WGS data of PC3, COLO320DM, and SNU16 cells and single cell ATAC-seq data of COLO320DM cells were collected with the source and ID numbers (Table S1). These cell lines have been validated for ecDNA regions (Table S2) [3, 9, 19].

### Data pre-processing

The sequence files in the fastq format were pre-processed and indexed using fastp [20], BWA [21], and SAMtools [22].

### ATACAmp pipeline

The ATACAmp workflow is shown in Fig. 1B and comprises the following steps.

#### Extracting abnormal reads

ATACAmp first transforms the BAM file obtained from the data pre-processing step into a Pysam object. Each read pair is interpreted to find the following abnormal reads.

(1) Split reads: Several parts of a read segment map to different reference genome regions, indicating that it may contain circular DNA breakpoints.

(2) Discordant reads: In paired-end sequencing, the sample DNA is first broken into fragments that are several hundred base-pairs in length and then sequenced from both ends of a fragment. In general, the length of the fragments will be in a limited range. Read pairs that map to a larger than normal region may indicate that certain structural variations, such as genomic rearrangements, have occurred in that region. For extrachromosomal circular DNA, the sequencing of fragments that covers the breakpoint from both ends can also result in abnormal intervals between paired reads. ATACAmp outputs these two types of reads into two types of BAM files.

#### Handling abnormal read segments

Based on the principle described above, split reads may contain circular DNA breakpoints, which can help locate the breakpoints. Discordant reads may also contain circular DNA breakpoints between them, thus containing a region that may contain breakpoints. ATACAmp processes the split and discordant reads obtained in the previous step separately as follows.

(1) In split reads, the circular DNA breakpoints include two positions on the reference genome. Based on the cigar value of a line in split BAM and the starting position compared with the reference genome, the breakpoint position on its side can be judged. BWA, when generating the comparison results, will compare two regions for a read segment in the “SA” tag, providing the information of the other comparison result. Using this information, the position of the other breakpoint side can be obtained from the “SA” tag and the orientation of the sequences on both breakpoint sides can be calculated. ATACAmp constructs a dictionary data structure with the chromosome and breakpoint position to save this information, and the breakpoint at the same position increases its support number, which can be a type of evidence of its real existence.

(2) For discordant reads, although the exact breakpoint position cannot be obtained, the number of discordant reads is much higher than that of split reads because it is not limited by the sequencing read length. For each read segment of an abnormal read segment pair, an interval of 500 bp upstream and downstream is constructed using the start site of their mapping to the reference genome. Once the construction is finished for all abnormal read segment pairs, any pairs wherein both segments are in the same interval are merged to create a new interval. This new interval is then used to move forward. After iteration, the construction of genome-wide region breakpoint pairs is complete, finally extracting the results to a file.

#### Finding amplification regions containing breakpoints

Because of the higher chromatin accessibility on circular DNA and multiple copies of circular DNA within the cell, ecDNA sequences should behave as contiguous regions of higher sequencing depth on ATAC-seq data. Therefore, ATACAmp was used to analyze the breakpoint pair regions identified from the abnormal read segments combined with their sequencing depths.

(1) First, the average sequencing depth is estimated for the whole genome using the Pysam package and the original BAM file; the subsequent threshold for determining whether to amplify is the “covt”. The breakpoint pairs identified from the discordant reads in the previous step are used as input and the breakpoint pairs are represented as an interval at both positions at this point. For this interval, the midpoint value is considered, and with this position as the starting point, the coverage upstream and downstream is slide-checked at 1000-bp intervals until both the total average coverage and average coverage of the new window are lower than the set threshold (covt), thereby completing the analysis of the continuous amplification region. These amplification regions are numbered, and the amplification region and length of the amplification region where each breakpoint position is located are marked in the breakpoint pair information. The breakpoint pairs where the amplification region is < 3000 are excluded.

(2) For the exact breakpoint location obtained from split reads, whether it is on the amplification region found in (1) is first determined. If so, it is marked directly, and if not, the same detection method as used in (1) is employed to find the amplification region where the breakpoint is located. If the amplification region length meets the condition, it is added to the breakpoint pair information obtained in (1).

(3) (1) and (2) breakpoint pairs are merged with the same amplification region at both locations in the breakpoint file.

#### Building a genome-wide collaborative amplification map

Using the above steps, ATACAmp obtains the amplification regions containing breakpoints across the whole genome. These regions are then used as nodes in a graph structure and the relationships between the breakpoint pairs form the edges. ATACAmp uses the Networkx package [23] to construct the graph structure and extracts different connected graphs within it, i.e., the co-amplified regions interconnected via breakpoints, using the graph theory algorithm of Networkx. For each connected graph, the loop structure may indicate that these structures are connected to form a circular DNA. If loops exist, ATACAmp outputs the loop nodes, identifies the maximum circle available to the user, and provides a visual structure of this connected graph via the Matplotlib module.

#### Annotating amplification region

ATACAmp employs an embedded functionality to annotate the amplification regions by integrating user-provided annotation files in the GTF format. This process involves associating annotations to the amplification regions that overlap with the corresponding gene regions specified in the GTF file.

#### Extracting cellular barcode in single-cell mode

Cells with abnormal read segments originating from the breakpoints on the amplification region were collected. Then, each cell was associated with the specific breakpoints on the amplification region that corresponded to its abnormal read segments.

### Single-cell ATAC clustering and labeling

A custom reference package for hg19 was established using cellranger-arc mkref. The ATAC-seq data were analyzed using ArchR [24]. Doublets were identified and excluded using ArchR. The ATAC-seq data dimensionality was reduced using iterative latent semantic indexing (LSI) with the addIterativeLSI function in ArchR. To estimate the accessibility gene scores, impute weights were incorporated using the addImputeWeights function and scores were visualized using the plotEmbedding function. Cell barcodes were used to mark the cells corresponding to the co-amplified region extracted from ATACAmp. The difference in MYC gene accessibility scores was calculated for this group of cells and the remaining cells. P-values were calculated using the Wilcoxon test.

### AmpliconAritect

The downloaded Fastq file is filtered for low quality Reads and bases using fastp software to remove splice sequences. Remove the Reads with length less than 50, and get the high quality Reads after filtering.

The reads from the previous step were compared to the reference genome hg38 using BWA software, and then converted into BAM files using the SAMtools view module. module to convert the generated SAM file into BAM format, and then use the SAMtools sort module to sort the reads according to their position in the reference genome. Use SAMtools sort module to sort the files according to the position of Reads comparison to the reference genome, and then get the sorted BAM files. Run PrepareAA in Docker and call AmpliconArchitect with default parameters.

### Circle-finder

After fastp preprocessing of ATAC-seq data for PC3, COLO320DM, SNU16 cell lines, the sequencing files were compared to the reference genome hg38 using BWA mem to obtain SAM files, and subsequently these BAM files were run Circle_finder-pipeline-bwa-mem-samblaster. sh with default parameters.

### Electronic supplementary material

Below is the link to the electronic supplementary material.

Supplementary Material 1

Supplementary Material 2

Supplementary Material 3

Supplementary Material 4

Supplementary Material 5

## Declarations

### Availability and requirements

Project name: ATACAmp.

Project home page: https://github.com/chsmiss/ATAC-amp

Operating system(s): Linux

Programming language: Python

License: MIT

Any restrictions to use by non-academics: licence needed

## Abbreviations

ecDNA: Extrachromosomal DNA
AA: AmpliconArchitect
HSR: Homogeneously staining regions
