# Supplementary material for: ATACAmp: a tool for detecting ecDNA/HSRs from bulk and single-cell ATAC-seq data
Source: BMC Genomics. 2023 Nov 10;24:678. doi: 10.1186/s12864-023-09792-6 (PMC10638764; doi:10.1186/s12864-023-09792-6)
Supplement: Supplementary file 4 — Supplementary Material 4 [file 12864_2023_9792_MOESM4_ESM.docx]

**Figure S1: PVT1 expression heterogeneity in the COLO320DM cell line.**

(A)The distributions of ecDNA positive cells in two-dimension UMAP space. (B) PVT1 accessibility scores were visualized on the ATAC–seq UMAP, showing cell-level heterogeneity in PVT1 ATAC-seq signals in COLO320DM. (C) Differences in accessibility scores of PVT1 gene between ecDNA positive and negative cells.

**Figure S2: MIR1205 expression heterogeneity in the colo320dm cell line.**

(A)The distributions of ecDNA positive cells in two-dimension UMAP space. (B) MIR1205 accessibility scores were visualized on the ATAC–seq UMAP, showing cell-level heterogeneity in MIR1205 ATAC-seq signals in COLO320DM. (C) Differences in accessibility scores of MIR1205 gene between ecDNA positive and negative cells.


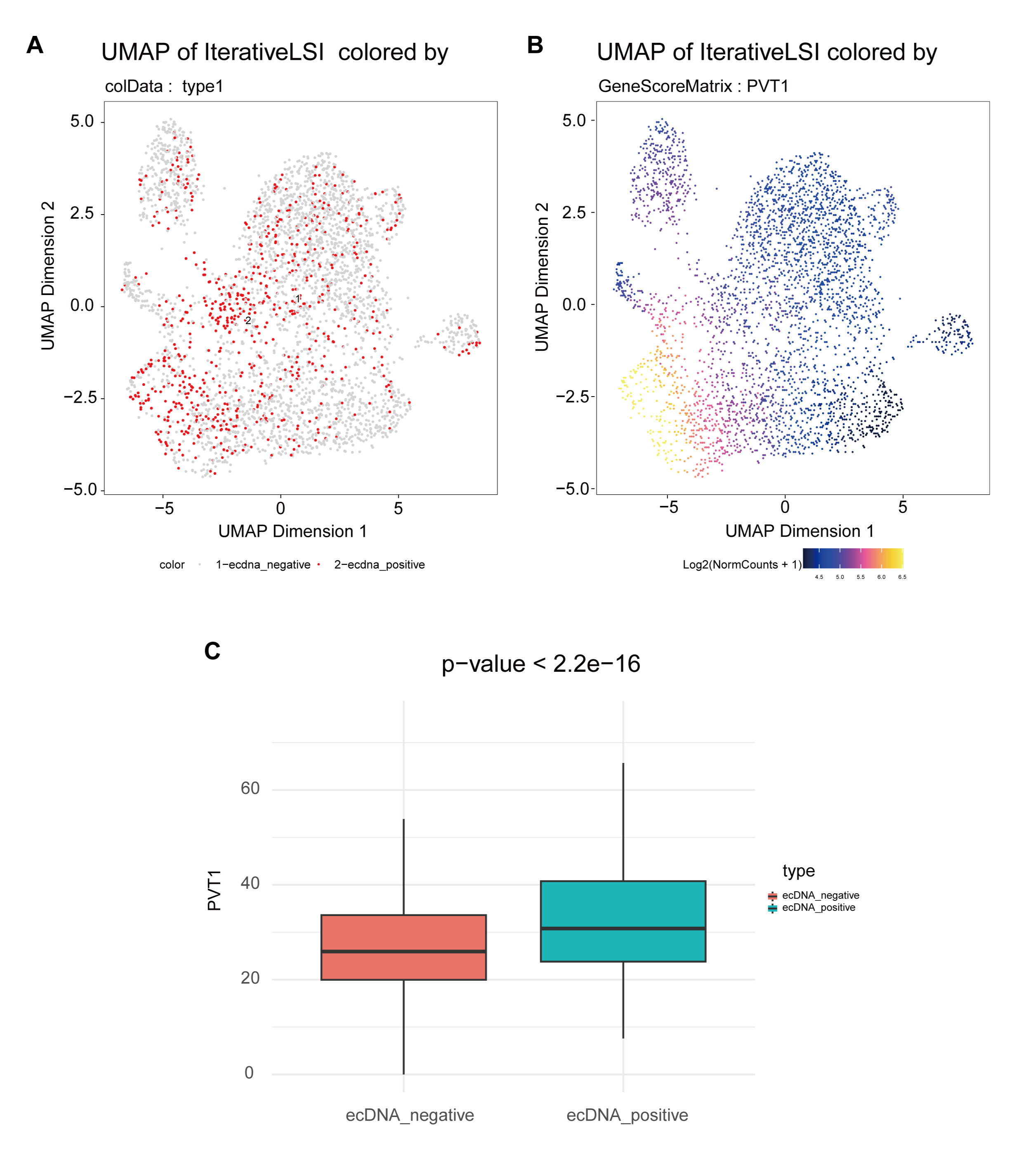


**Figure S1:PVT1 expression heterogeneity in the COLO320DM cell line.**


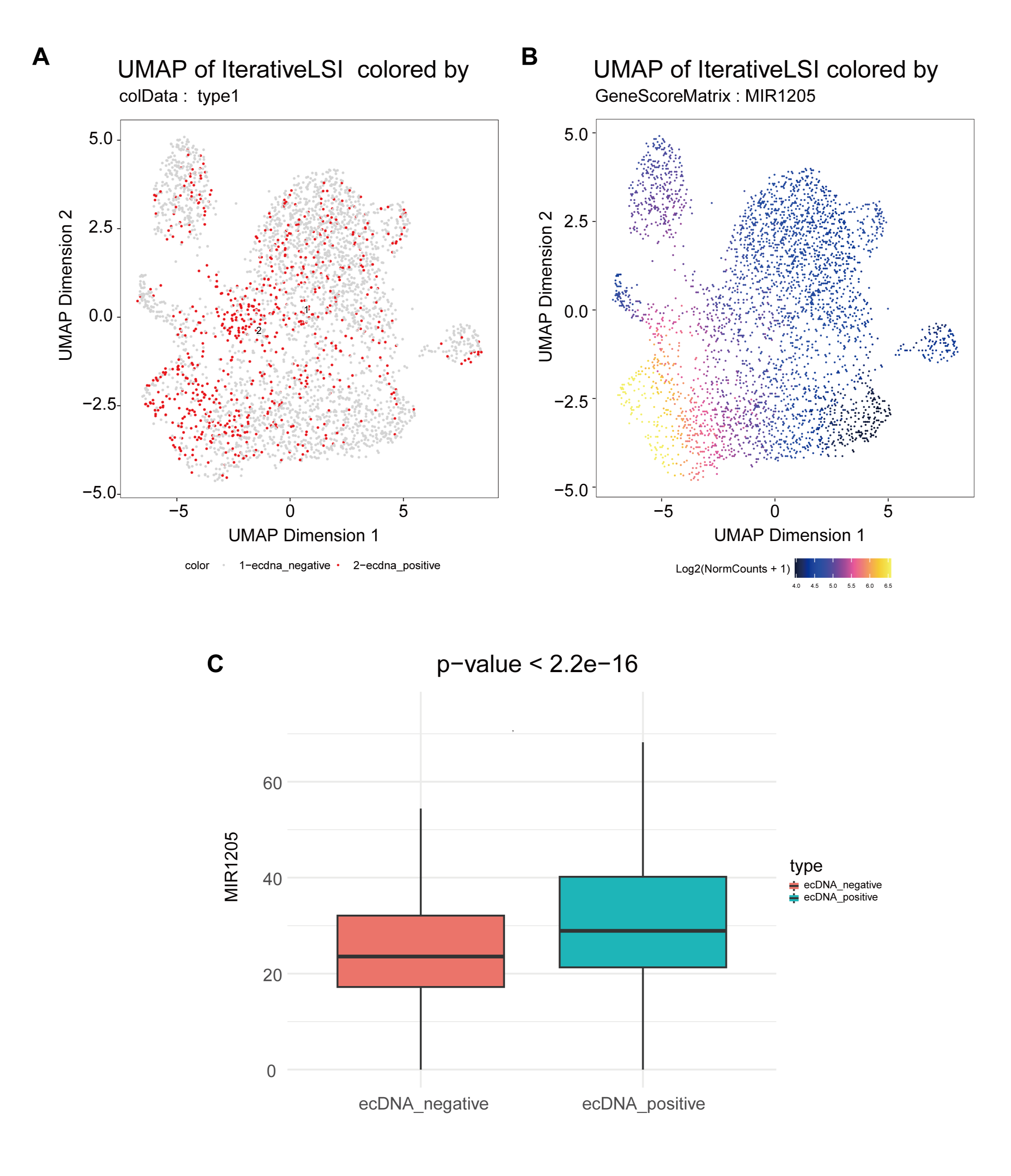


**Figure S2: MIR1205 expression heterogeneity in the colo320dm cell line.**
