# Supplementary material for: ATACAmp: a tool for detecting ecDNA/HSRs from bulk and single-cell ATAC-seq data
Source: BMC Genomics. 2023 Nov 10;24:678. doi: 10.1186/s12864-023-09792-6 (PMC10638764; doi:10.1186/s12864-023-09792-6)
Supplement: Supplementary file 5 — Supplementary Material 5 [file 12864_2023_9792_MOESM5_ESM.docx]

**Supplementary Information**

**[Additional file 1: Table S1.](https://static-content.springer.com/esm/art:10.1186/s12864-023-09546-4/MediaObjects/12864_2023_9546_MOESM3_ESM.xlsx)**

Data collection for testing.

**[Additional file 2: Table S2.](https://static-content.springer.com/esm/art:10.1186/s12864-023-09546-4/MediaObjects/12864_2023_9546_MOESM3_ESM.xlsx)**

ecDNA composition detected using different methods.

**[Additional file 3: Table S3.](https://static-content.springer.com/esm/art:10.1186/s12864-023-09546-4/MediaObjects/12864_2023_9546_MOESM3_ESM.xlsx)**

AmpliconArchitect and ATACAmp performance evaluation

**Additional file 4:**

Figure S1: PVT1 expression heterogeneity in the COLO320DM cell line.

Figure S2: MIR1205 expression heterogeneity in the COLO320DM cell line.
